# The Amazing World of IDPs in Human Diseases II

**DOI:** 10.3390/biom12030369

**Published:** 2022-02-25

**Authors:** Simona Maria Monti, Giuseppina De Simone, Emma Langella

**Affiliations:** Institute of Biostructures and Bioimaging, CNR, Via Mezzocannone 16, I-80134 Naples, Italy; giuseppina.desimone@cnr.it

Intrinsically Disordered Proteins (IDPs) lack stable tertiary and secondary structures and are extensively distributed across eukaryotic cells, playing critical roles in cell signaling and regulation [1,2,3]. IDPs are also frequently associated with the development of diseases such as cancer, cardiovascular diseases, and neurodegenerative diseases [4,5,6,7]. For this reason, they have been converted into attractive therapeutic targets, although targeting them is challenging due to their dynamic nature. Indeed, the structural flexibility of IDPs causes difficulties in reliably capturing their heterogeneous structures through conventional experimental methods; thus, new methods and approaches have been developed [8]. This Special Issue includes seven articles from more than 35 scientists around the world working in the amazing field of IDPs. The contributions illustrate the most recent progress in knowledge on IDPs and human diseases.

The Special Issue begins with the article by Atieh and colleagues [9], which describes an interesting investigation on α-synuclein (αSyn) and DJ-1, an antioxidative protein that plays a critical role in Parkinson’s disease (PD) pathology. Through nuclear magnetic resonance (NMR) spectroscopy integrated with atomic force microscopy (AFM) in solution, the authors characterized the interaction of DJ-1 with glycated N-terminally acetylated-αSyn (glyc-ac-αSyn). The obtained results show that DJ-1 interacts with glycated and native ac-αSyn through the catalytic triad and establish that the oxidation state of the catalytic cysteine is imperative for binding. A mechanism of action by which DJ-1 interacts with N-terminally acetylated-αSyn oligomers, preventing their interaction with glyc-ac-αSyn monomers, was proposed. The relevance of these results within PD pathology shows how DJ-1 function in chaperoning αSyn may prevent the rapid accumulation of aggregated αSyn within the cell, which may enable proper clearance mechanisms from the cell and reduce the effects of neurodegeneration. Therapeutics targeting the effects of glycation in conjunction with maintaining proper DJ-1 function may mitigate neurodegeneration and diminish the symptoms of PD.

Another remarkable study within this Special Issue was carried out by Rizzuti and coworkers [10] on nuclear protein 1 (NUPR1), which is a small, highly basic ID protein of 82 residues that localizes throughout the whole cell, and is involved in the development and progression of several tumors. Based on previous results, the authors designed and synthetized nine derivatives, starting from lead compound ZZW-115, which were then investigated through biophysical and cellular experiments. Interestingly, the authors highlight how a more favorable binding affinity does not necessarily correlate with biological effects, underlining the importance of having a subtle compromise between increasing drug affinity and altering protein function, in addition to other properties such as solubility, crowding, membrane permeation, cellular efflux and cellular metabolism.

Cardone and co-workers thoroughly investigated phosphoprotein P of Mononegavirales (MNV) [11], which is an essential co-factor of the viral RNA polymerase L and whose prime function is to recruit L to the ribonucleocapsid composed of the viral genome encapsidated by the nucleoprotein N. The authors investigated the dynamic behavior of P_Cα_, a domain that is C-terminal to the small oligomerization domain (P_OD_) and constitutes the respiratory syncytial virus L-binding region together with P_OD_. By using small phosphoprotein fragments centered on or adjacent to P_OD_, a structural picture of the P_OD_–P_Cα_ region in solution was gained, evidencing how small molecules are able to modify the dynamics of P_Cα_. This observed structural plasticity of the P_Cα_ domain may play a crucial role for the functional viral polymerase, which needs more investigations.

The paper by Ortega-Alarcon and colleagues [12] investigated methyl-CpG binding protein 2 (MeCP2), a multidomain IDP associated with neuronal development and maturation. In particular, the authors focused their attention on MBD, one of the key domains in MeCP2 responsible for DNA recognition, and its two flanking disordered domains, NTD and ID. It was demonstrated that both the disordered domains—NTD and ID—unequivocally stabilize the MBD domain against thermal and chemical denaturation and that NTD-MBD-ID differs functionally and structurally from MBD. The authors also highlight how disorder in proteins may be considered a pervasive feature that is even more important in multidomain IDPs with a complex conformational and multifunctional landscape.

The structural features of FOXO3 were analyzed by Weinzierl [13] through multiple independent molecular dynamics simulations of models of full-length FOXO3 bound to DNA, using both implicit and explicit solvation conditions. FOXO3, belonging to the ‘forkhead box O’ gene family, is of considerable interest in many therapeutically relevant areas, such as tumor therapy and longevity research. The obtained results provide atomistic models for an extended structure of FOXO3 when bound to DNA, showing that the two ‘linker’ regions immediately adjacent to the DNA-binding domain are present in a highly extended conformation, likely due to electrostatic repulsion of the domains connected by the linkers. The study sheds light on previously unrecognized structural properties of FOXO3 and introduces a new graphical method of general use that is particularly helpful for studying and visualizing the structural diversity of IDPs.

Bokor and Tantos [14] studied two different IDP interaction systems to gain information about the bonds holding the protein associations together using wide-line 1H NMR. One system consisted of wild type and mutant α-synuclein (αS) in the forms of oligomers and amyloids and the other system was the complex between the intrinsically disordered (IDP) thymosin-β4 (Tβ4) and the cytoplasmic domain of stabilin-2 (stabilin CTD), which is involved in the phagocytosis of apoptotic cells. The study provides insights into the intermolecular bonds that contribute to the formation of αS oligomer and amyloid aggregates. Moreover, the authors revealed information on the molecular background of the fuzzy complexes between thymosin-β4 and stabilin-2 CTD.

The work presented by Raut and colleagues [15] investigated Par-4 (Prostate apoptosis response-4), a predominantly intrinsically disordered protein, acting as a tumoural suppressor that is capable of selectively inducing apoptosis in cancer cells while leaving healthy cells unaffected. The authors performed experimental studies, employing circular dichroism spectroscopy and dynamic light scattering to assess the effects of various monovalent and divalent salts upon the conformation of cl-Par-4 in vitro. The obtained results clarify the different roles of cations and anions in influencing Par-4 structure and indicate that the SAC domain of the protein, which is the region of Par-4 indispensable for its apoptotic function, is likely to be helical in cl-Par-4 under the studied high salt conditions.

In summary, the papers collected in this Special Issue unveil novel aspects related to the wide world of IDPs. Using a variety of methods, including biochemical, spectroscopic and computational techniques, they represent a step forward in the study and characterization of many IDPs involved in human diseases, with a focus on conformational features, environmental effects, recognition mechanisms and targeting.
